# S03-1 Falls Management Exercise for those at high or intermediate risk of falls. Improving habitual physical activity, function and reducing falls

**DOI:** 10.1093/eurpub/ckae114.207

**Published:** 2024-09-26

**Authors:** Dawn Skelton

**Affiliations:** Research Centre for Health (ReaCH), School of Health and Life Sciences, Glasgow Caledonian University, Glasgow, United Kingdom

## Abstract

**Purpose:**

FaME, a group based multi-component exercise programme, was developed by researchers to reduce the risk of falls and help participants regain confidence and self-efficacy to increase physical activity, as fallers often avoid activity through concern about falls. Uniquely, FaME includes retraining the ability to get up from the floor, reducing fear of falling and potential ambulance call outs for participants. The original FaME RCT (history of frequent falls, 9mths programme) and the ProAct65+ RCT (sedentary older people, 6mths programme) showed reduced falls and improved habitual physical activity (extra 105 mins MVPA/wk), even 24 months post intervention. These benefits have been seen in robust randomised controlled trials, service evaluations and peer-reviewed published ‘real-life’ delivery across the UK. It has been adapted as an EXERGAME App, and a dance intervention by researchers and organisations.

**Project description:**

FaME is delivered by trained Postural Stability Instructors (PSIs) and is cited as a cost-effective exercise programme by the Department of Health and Social Care, Public Health England, Public Health Wales, the Scottish Government, the Royal College of Physicians, the Royal Osteoporosis Society, the Chartered Society of Physiotherapy, the Centre for Ageing Better, the Royal Society for the Prevention of Accidents and Age UK. Outside of the UK, FaME is embedded in the Norwegian Health Ministry programme Sterk Og Stødig, a case study in the World Health Organisation’s Step Safely Report, an effective intervention endorsed by the Centre for Disease Prevention in the US and is recommended in the World Falls Guidelines. Implementation studies are examining how scaling up has led to adapted delivery which does not always meet key fidelity parameters for effectiveness (duration, dose, components included, equipment, progression, and transition). Regular CPD offered to PSIs and a community of practice can support real life implementation.

**Conclusions:**

FaME is the first falls prevention exercise programme to show sustained positive effects up to 2 years post intervention on habitual physical activity. The rollout of FaME has the potential to change the activity and frailty trajectory of older people with a history of falls.

